# Stepwise Toward Pure Blue Organic Light‐Emitting Diodes by Synergetically Locking and Shielding Carbonyl/Nitrogen‐Based MR‐TADF Emitters

**DOI:** 10.1002/advs.202401664

**Published:** 2024-05-05

**Authors:** Jie‐Rong Yu, Hong‐Ji Tan, Xiu‐Qi Gao, Bing Wang, Zhi‐Qiang Long, Jia‐Li Liu, Zhi‐Zhong Lin, Xing‐Yi Li, Ze‐Lin Zhu, Jing‐Xin Jian, Qing‐Xiao Tong, Chun‐Sing Lee

**Affiliations:** ^1^ College of Chemistry and Chemical Engineering Key Laboratory for Preparation and Application of Ordered Structural Material of Guangdong Province Shantou University Shantou 515063 P. R. China; ^2^ Center of Super‐Diamond and Advanced Films (COSDAF) and Department of Chemistry City University of Hong Kong Hong Kong SAR 000000 P. R. China; ^3^ Department of Chemistry City University of Hong Kong Hong Kong SAR 000000 P. R. China

**Keywords:** deep‐blue, MR‐TADF, OLEDs, shielding

## Abstract

Deep‐blue multi‐resonance (MR) emitters with stable and narrow full‐width‐at‐half‐maximum (FWHM) are of great importance for widening the color gamut of organic light‐emitting diodes (OLEDs). However, most planar MR emitters are vulnerable to intermolecular interactions from both the host and guest, causing spectral broadening and exciton quenching in thin films. Their emission in the solid state is environmentally sensitive, and the color purity is often inferior to that in solutions. Herein, a molecular design strategy is presented that simultaneously narrows the FWHM and suppresses intermolecular interactions by combining intramolecular locking and peripheral shielding within a carbonyl/nitrogen‐based MR core. Intramolecularly locking carbonyl/nitrogen‐based bears narrower emission of 2,10‐dimethyl‐12,12‐diphenyl‐4H‐benzo[9,1]quinolizino[3,4,5,6,7‐defg]acridine‐4,8(12H)‐dione in solution and further with peripheral‐shielding groups, deep‐blue emitter (12,12‐diphenyl‐2,10‐bis(9‐phenyl‐9H‐fluoren‐9‐yl)−4H‐benzo[9,1]quinolizino[3,4,5,6,7‐defg]acridine‐4,8(12H)‐dione, DPQAO‐F) exhibits ultra‐pure emission with narrow FWHM (c.a., 24 nm) with minimal variations (∆FWHM ≤ 3 nm) from solution to thin films over a wide doping range. An OLED based on DPQAO‐F presents a maximum external quantum efficiency (EQE_max_) of 19.9% and color index of (0.134, 0.118). Furthermore, the hyper‐device of DPQAO‐F exhibits a record‐high EQE_max_ of 32.7% in the deep‐blue region, representing the first example of carbonyl/nitrogen‐based OLED that can concurrently achieve narrow bandwidth in the deep‐blue region and a high electroluminescent efficiency surpassing 30%.

## Graphical abstract

Synergistic effect of intermolecular locking and peripheral shieling endows carbonyl/nitrogen‐based multi‐resonance thermally activated delayed fluorescent emitter with the narrow spectrum and tiny emission broadening (≤ 3 nm) in a thin film over a wide doping concentration, presenting cutting‐edge performance in deep‐blue device with external quantum efficiency surpassing 32%.

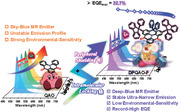

## 1 Introduction

Pioneering research by Hatakeyama and co‐worker in 2016 presented co‐doping electron‐rich/‐deficient atoms into polycyclic aromatic hydrocarbons (PAHs), resulting in frontier orbitals alternatively centered in electron‐rich/‐deficient atoms and their respective resonant atoms.^[^ ^1^ ^]^ This approach successfully narrows the energy offset of S_1_ and T_1_ states, leading to thermally activated delayed fluorescence in PAHs. Thus, these PAHs are named multi‐resonant (MR‐TADF) emitters.^[^ ^2^, ^3^ ^]^ The rigid structure and short‐range charge transfer (SRCT) feature endows MR‐TADF organic light‐emitting diodes (OLEDs) with high efficiency and narrowband emission (high color purity).^[^ ^4^, ^5^, ^6^ ^]^

A narrowband emission is highly desired for blue light as it requires a lower energy gap to achieve similar Commission Internationale d'Eclairage (CIE) y coordinates. This allows for greater flexibility in host material selection, reduces carrier injection barriers,^[^ ^7^, ^8^ ^]^ and increases the possibility of achieving high performance and stability in devices. For instance, DBCz‐Mes^[^ ^9^ ^]^ (Scheme S1, Supporting Information) with lower energy gap and emission peak/full width at half maximum (FWHM) of 452/17 nm (CIEy = 0.058) in device has comparable CIEy with NOBNacene^[^ ^10^ ^]^ (409/37 nm, CIEy = 0.055) and MesB‐DIDOBNA‐N^[^ ^11^ ^]^ (405/31 nm, CIEy = 0.055), counterparts with much higher energy gap with peaks/FWHMs. Extremely, a line emission at 467 nm is with CIEy < 0.05.^[^ ^12^ ^]^ To narrow down emission FWHM, Hatakeyama et al. presented the extension of π‐conjugation from DABNA‐1 to ν‐DABNA, which resulted in an enhanced resonant effect and reduced the FWHM from 33 to 14 nm in toluene.^[^ ^1^, ^4^ ^]^ Similar research using multi‐boron doping or replacing N with a stronger electron‐donating atom to enhance the resonant effect of DABNA‐1 or BCzBN^[^ ^6^ ^]^ evidenced the success of this strategy.^[^ ^13^, ^14^ ^]^ Alternatively, increasing the rigidity of parental compound DABNA‐1 or BCzBN can restrain molecular bending and rocking, effectively narrowing emission profiles. Lee et al., Duan et al., and Jiang et al. applied this strategy to QAO and BCzBN to narrow the FWHM from > 30 nm to < 15 nm in toluene.^[^ ^9^, ^15^, ^16^ ^]^ However, these rigid and large planar PAHs are prone to interact with their surrounding molecules, resulting in emission redshifting and/or broadening, and even quenching, which ultimately leads to unsatisfied color purities and electroluminescent (EL) in devices.^[^ ^17^, ^18^, ^19^, ^20^ ^]^ Yang et al. and Duan et al. independently reported on carbazole‐shielded BCzBN derivatives presenting sky‐blue emissions with ultrahigh performance (EQE > 35%) and narrow FWHM over wide doping ranges (c.a. ≈30 wt.%).^[^ ^19^, ^20^ ^]^ Jiang et al. pointed out that without *tert*‐butyl substituents, CZO showed much inferior color and efficiency compared to CZ2CO (FWHM: 63 vs 23 nm; EQE 10.3% vs 13%) in the device.^[^ ^16^ ^]^ Even with triple *tert*‐butyl shielding, the FWHM of CZ2CO is broadened from 16 nm in toluene to 23 nm in the device. Thus, retaining FWHMs and efficiencies of MR blue emitters in the solid state is necessary and challenging. This is particularly vital for MR‐systems (e.g., QAO) featuring a large molecular polarity index (MPI, Figure S1, Supporting Information)^[^ ^21^, ^22^ ^]^ as the electrostatic interactions of the polarized groups will induce close alignments between adjacent molecules for stabilizing the thermodynamic energy of the system.

To retain narrow FWHM and high efficiency of MR blue emitters in the solid state, environmental factors (including guest‐guest and host‐guest interactions) like π–π interactions, dipole‐dipole interactions, etc., should be suppressed.^[^ ^15^ ^]^ Spatial shielding of emitting cores has been successfully achieved in sky‐blue and green emitters.^[^ ^2^, ^12^ ^]^ For blue MR emitters, an ideal shielding group should be 1) bulkier than conventional steric groups (e.g.*, tert*‐butyl, phenyl, adamantanyl, mesityl, etc.); 2) with high triplet energy (*E*_T_); and 3) electronically inert to the MR system. The first requirement ensures that the MR cores are appropriately protected, while the second requirement is to prevent the draining of triplet excitons without undermining the emission efficiency of the cores. The third requires such groups not to interrupt the SRCT feature or significant red‐shift emission of MR molecules, maintaining efficient narrowband blue emissions of the MR cores. This point requires extra caution in modifying the MR system as a phenyl group extended in BCzBN leads to green emission and weak donors like 1,3,6,8‐tetramethyl‐9*H*‐carbazole shifts QAO to a pull‐push CT system.^[^ ^23^, ^24^, ^25^ ^]^

In this study, we demonstrated effective retention of narrow blue emission in solid film for a carbonyl/nitrogen MR‐system, QAO, by locking and shielding the core step by step. First, the FWHM of QAO is relatively large (32 nm)^[^ ^26^ ^]^ compared to BN‐based MR cores, and its “bay area” (C4‐N3‐C15 in the diphenylamine‐like unit, Figure S2, Supporting Information) was locked with a diphenylmethanyl (DP) bridge, giving *Locked*‐QAO (*L*‐QAO): 2,10‐dimethyl‐12,12‐diphenyl‐4*H*‐benzo[9,1]quinolizino[3,4,5,6,7‐*defg*]acridine‐4,8(12*H*)‐dione (DPQAO‐M) with a more rigid structure. Next, a novel shielding group 9‐phenyl‐9*H*‐fluoren‐9‐yl (PF, Figure S3, Supporting Information) units were attached to QAO by connecting to the sp^3^ carbon, bearing locked and shielded QAO derivatives, 12,12‐diphenyl‐2,10‐bis(9‐phenyl‐9*H*‐fluoren‐9‐yl)−4*H*‐benzo[9,1]quinolizino[3,4,5,6,7‐*defg*]acridine‐4,8(12*H*)‐dione (DPQAO‐F). Compared to QAO, the DP‐locked compound (DPQAO‐M) exhibited narrower blue emission in solution (32 vs 24 nm) while its FWHM was widened to > 30 nm in host‐containing films. In contrast, the PF‐shielded *L*‐QAO (DPQAO‐F) inherited narrowband emission of *L*‐QAO in solution (24 vs 23 nm) and retained the emission peak position and FWHM under thin films state with a tiny variation of FWHM as doping ratio up to 8 wt.% (∆FWHM ≤ 3 nm). Concomitantly, the TADF‐OLED based on DPQAO‐F shows ultra‐pure deep‐blue emission with λ_EL_/FWHM = 463/24 nm and comparable EQE values of 17.9%–19.9% over a wide doping range of 1–8 wt.%, evidencing a property of anti‐quenching, ‐redshifting and ‐spectral broadening. In a hyperfluorescent (HF) OLED using DPQAO‐F as a dopant emitter, excellent EQE up to 32.7% with a CIE_y_ coordinate of 0.117 was achieved. To the best of our knowledge, it is the first example of QAO‐based OLEDs simultaneously achieving deep‐blue emission and EQE value of over 30%. This work reveals that effectively suppressing guest‐guest and host‐guest interactions can effectively retain narrowband deep‐blue emission in the solid state, which enhances the practical reference of MR emitter design.

## 2 Results and Discussion

### 2.1 Theoretical Calculation and Synthesis

**Figure 1:**
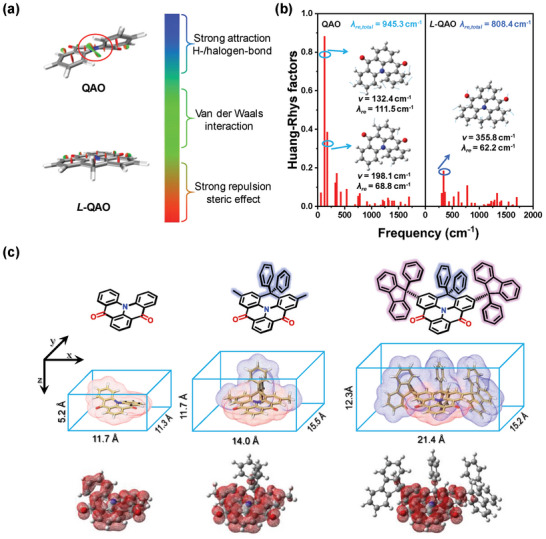
a) reduced density gradient (RDG) analysis between QAO and *L*‐QAO. b) Calculated Huang‐Rhys (HR) factors and vibration frequency *v* and reorganization energy *λ_re_* of the S_1_‐S_0_ transition of QAO and *L*‐QAO. c) The chemical structures (top), calculated length, width, and height (middle), and triplet spin‐density distributions (bottom) of the optimized molecular geometry of QAO, DPQAO‐M, and DPQAO‐F based on b3lyp/6‐31G (d, p) level.

The calculated S_1_‐S_0_ transition and spin‐density distribution (SDD) of T_1_ states are mainly located in the carbonyl/nitrogen emitting core (**Figure** **1**; Figure S4, Supporting Information), and thus, the QAO core governs the molecular emission properties. QAO is noted to have an obvious distortion in the “bay area” due to steric repulsion between the C─H bond of C4 and C15. The DP locking in *L*‐QAOs (DPQAO‐M and DPQAO‐F) will cancel this repulsion and further planarize the molecular skeleton.^[^ ^9^, ^27^, ^28^ ^]^ To further unveil the underlying factors, we performed a reduced density gradient (RDG) analysis between QAO and the *L*‐QAO (all peripheral groups are removed for better clarity and convergence). As expected, the calculation results confirm that QAO has strong repulsion between adjacent C─H bonds in the “bay area” compared to *L*‐QAO (Figure 1a). Consistently, a larger Huang–Rhys (HR) factor, vibration frequency (*v*), and reorganization energy (*λ*_re_) of the S_1_‐S_0_ transition were found in QAO, which were origin from the twisting vibrations mode in 132.4 and 198.1 cm^−1^ and corresponding to the strong out‐of‐plane bending vibration of resisting unit with ***λ*** of 111.5 and 68 cm^−1^ (Figure 1b). In contrast, only stretching vibrations were found in *L*‐QAO (***v*** and ***λ*** of 355.8/62.2 cm^−1^), reducing the total *λ*_re_ of 808.4 cm^−1^ for *L*‐QAO (vs 945.3 cm^−1^ of QAO). The smaller HR factor, less vibrations mode, and reduced *λ*_re_ will contribute to narrowing the spectrum of free molecules (i.e., in solution). Next, the shielding effect of PF in *L*‐QAO was further explored by calculating their geometrical electronic structures, and the results were depicted in Figure 1c. As the bulky substituents (blue area) increase from QAO, DPQAO‐M to DPQAO‐F, the molecular dimensions in *x, y, and z* axes increase from 11.7/11.3/5.2 to 21.4/15.2/12.3 Å, and the blue gradually wraps the red area (MR‐core). Compared to the bare QAO‐core (^3^LE = 2.55 eV),^[^ ^23^ ^]^ QAO‐core in DPQAO‐F is surrounded by PF moieties with a high triplet energy (^3^LE = 2.88 eV, Figure S5c, Supporting Information), which defense triplet exciton against intermolecular interactions and diffusing (triplet‐triplet annihilation and Dexter energy transfer).

DPQAO‐M and DPQAO‐F were synthesized using a three‐step route, including the Ullman reaction, hydrolysis reaction, and Friedel–Crafts acylation. Structures of the target compounds were unambiguously confirmed with ^1^H and ^13^C NMR spectra, matrix‐assisted laser desorption/ionization coupled time‐of‐flight mass spectrometry (MALDI‐TOF), and single‐crystal X‐ray structure analysis. Details of these characterizations and the synthesis are presented in (Scheme S2, Supporting Information).

### 2.2 Photophysical Properties

**Figure 2:**
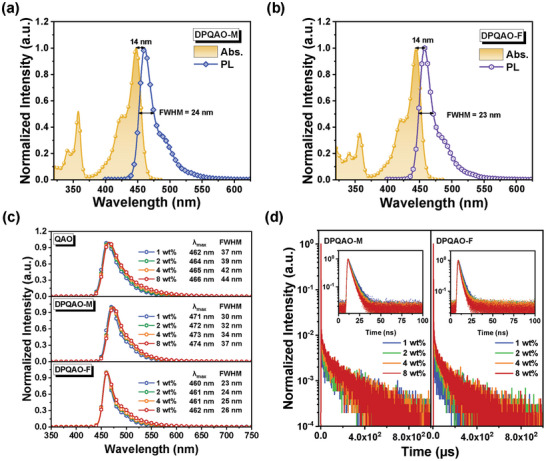
Normalized UV–vis absorption and fluorescence spectra of a) DPQAO‐M and b) DPQAO‐F in diluted toluene solutions (10^−5 ^M). c) Steady‐state PL spectrum of QAO, DPQAO‐M, and DPQAO‐F doped in 1, 2, 4, 8 wt.% mCP film at 300K. d) Transient PL curves for DPQAO‐M and DPQAO‐F.

Molecular photophysical properties of the DPQAO‐M and DPQAO‐F were investigated in diluted toluene solutions (10^−5^ m) at room temperature and presented in **Figure** **2**a,b and S1 (Supporting Information). Two compounds shared similar UV–vis absorption and emission profiles as predicted by theoretical calculations. The π–π^*^ absorption bands occurred at ≈350 nm, while the intense and sharp absorption peaking at 444 and 447 nm were ascribed to the characteristic SRCT transition of MR‐TADF emitters. Both DPQAO‐M and DPQAO‐F possessed intense mirroring emission located at 461 and 458 nm with small stokes shifts (both 14 nm), indicating that the introduction of the steric group does not result in increased structural relaxations during the excitation‐emission process. In comparison to QAO, both DP‐lock MR emitters show narrower FWHMs (32, 24, and 23 nm for QAO,^[^ ^26^ ^]^ DPQAO‐M, and DPQAO‐F), which is highly consistent with the expectation of the molecular design. In solvatochromic experiments, both DPQAO‐M and DPQAO‐F showed positive solvatochromic effects with 43 and 35 nm bathochromic shifts and widening of FWHMs by varying the solvent polarities from n‐hexane to dimethylsulfoxide (Figure S7, Supporting Information). The less bathochromic shift in DPQAO‐F is ascribed to the bulkier molecule, weakening the effect of the dipole field of surrounding solvent molecules.

The shielding effect of the PF group in the solid state was investigated via the concentration‐dependent PL properties of the three molecules in 1,3‐Di(9*H*‐carbazol‐9‐yl)benzene (mCP) films with a doping ratio of 1, 2, 4, and 8 wt.%(Figure 2c), respectively. Compared to the solution state, QAO shows a similar emission peak (466 vs 462 nm) but increased spectral FWHM (32 vs 37 nm) in the emission profile even at a very low doping of 1 wt.%. DPQAO‐M with DP‐lock shows reduced FWHMs (24 nm) in solution but noticeable FWHM widening (31 nm) at 1 wt.% host‐containing film. In contrast, the PF‐shielded DPQAO‐F‐based blend shows ultra‐pure deep‐blue emission peaking at 460 nm and narrow FWHM of 23 nm consisting of its solution PL. As doping concentration goes up, a very consistent emission profile with tiny ∆FWHM and ∆*λ*_em_ of 3 and 2 nm was observed in DPQAO‐F‐based films. In addition, the effectiveness of PF groups in shielding host‐guest interactions is evaluated using both p‐type and n‐type host media (including CzSi, mCP, TSPO1, and DPEPO; Figure S8, Supporting Information). Without shielding, QAO and DPQAO‐M show widened FWHM ranging over 42–48 and 34–46 nm, respectively. DPQAO‐F maintains the narrowest FWHM (25–37 nm) in these media. A large increase in PL quantum yield (*Φ*_PL_) from solution (32.3 and 34.1% for DPQAO‐M and DPQAO‐F, respectively) to mCP film (2 wt.%) was also observed, which can be attributed to the activation of delayed fluorescence in the dope films (Figure 2d; Figure S5 and Table S2, Supporting Information). DPQAO‐M and DPQAO‐F have no delayed emission signal in solution but show obvious delayed tails when doped in mCP. Temperature‐dependent transient PL decay spectra were measured and depicted in Figure S10c,d (Supporting Information). Both delayed signals of DPQAO‐M and DPQAO‐F showed apparent thermally activated trends with the increasing temperature, illustrating the accelerated RISC process. The small change of Δ*E*_ST_ from solution (0.25 and 0.24 eV, Figure S5a,b, Supporting Information) to mCP film (0.22 and 0.21 eV, Figure S10a,b, Supporting Information) should not be the reason for the absence of the TADF process in solution and an “exciplex‐like” host‐guest interaction is revealed as the origin of delayed fluorescence, as a similar phenomenon is reported by Chou et al.^[^ ^15^, ^29^, ^30^ ^]^ As shown in Figure S13 (Supporting Information), DPQAO‐F bears narrower Δ*E*_ST_ compared with that of DPQAO‐M (0.33 vs 0.40 eV), which contributes to a shorter observed delayed emission lifetime (τ_d_) in DPQAO‐F‐based films (i.e., 125.44 vs 163.64 µs in 2 wt.% blends, Table S2, Supporting Information). In these mCP blends, DPQAO‐F‐based films deliver higher *Φ*_PL_ and shorter τ_d_ with a narrow variation range of 84.2–77.9% and 125.44–107.54 µs than those of DPQAO‐M (77.7–56.9% and 189.96–133.21 µs), suggesting the PF group plays a role against concentration quenching. These PL results reveal the effectiveness of the PF group in protecting the MR emitting core from intermolecular interactions (both between guest molecules and between host‐guest molecules) and the inertness of the PF group to both the MR emitting core and surrounding molecules.

### 2.3 Single Crystal Analysis

**Figure 3:**
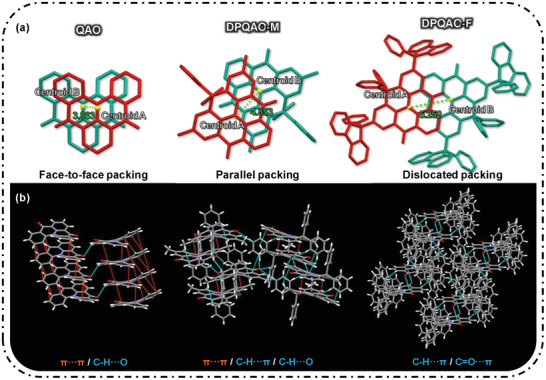
a) Centroid distance between the closest dimer and b) main interaction between QAO, DPQAO‐M, and DPQAO‐F.

To gain better insight into the impact of intramolecular lock modifications and peripheral steric units, single crystals of QAO, DPQAO‐M, and DPQAO‐F were grown by diffusing acetonitrile into their dichloromethane solutions. As shown in Figure S14 (Supporting Information), the monomolecular structure for three emitters is highly coincidental with the theoretical prediction, wherein the DP‐lock is almost orthogonal to the MR‐core and provides a moderate hindrance effect for DPQAO‐M. On the other hand, a large twisted angle ≈67° was found between two attaching PF groups and QAO‐core in the DPQAO‐F, showing obvious bulkier in three dimensions. The close packing distances between the N/C═O‐core (emitting‐core) were evaluated by the centroid distance of the face‐to‐face packing dimer, as shown in **Figure** **3**a. QAO, DPQAO‐M, and DPQAO‐F have centroid distances of 3.863, 4.853, and 5.252 Å and their MR‐cores are gradually glided away with decreasing co‐facing area. From QAO, DPQAO‐M to DPQAO‐F, effective π–π interaction (orange line) is decreasing to disappearance (Figure 3b) and CH‐π/CH‐O/CO‐π interactions aid in crystal packing. The increasing intermolecular distances in the three molecules imply a looser packing with weaker interactions intermolecularly, explaining DPQAO‐F's superior color purity and efficient deep‐blue emission in solid.

### 2.4 Molecular Dynamics Analysis

**Figure 4:**
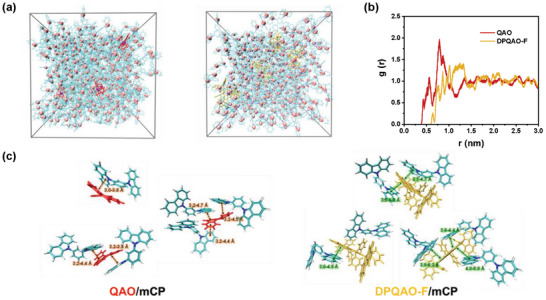
a) MD simulation results of the mCP: QAO/DPQAO‐F host‐guest system with a molar ratio of 294:6, corresponding to the 2 wt.% doped mCP film. (The QAO, DPQAO‐F, and mCP were shown in red, gold, and cyan, respectively. b) RDFs between COMs of emitting‐core for QAO/DPQAO‐F and Cz unit of mCP (shown as purple and pink balls). c) The intermolecular distances between guest and host molecules (three pairs of molecules with closest distances were selected).

Molecular‐dynamics (MD) simulations were performed on 2 wt.% QAO and DPQAO‐F (stands for two extreme examples) in mCP to better understand the guest‐host interaction of these molecules with surrounding host molecules. **Figure** **4**a shows the center of masses (COMs) of emitting‐core for QAO/DPQAO‐F and Cz (carbazole) unit of mCP, and the radial distribution functions (RDFs, g(r)) of the COMs calculated, which is used to measure the probability of finding a COM at a distance from another reference COM (Figure 4b).^[^ ^31^ ^]^ In the MD box of QAO, Cz units appear around QAO in a very near distance of 3.8 Å and the possibility continuously increases from 3.8 to 5.7 Å region around QAO, which facilitates close intermolecular interactions (e.g., co‐face π‐interactions, dipole‐dipole interactions) to happen. In contrast, the nearest Cz was found with a distance > 6.0 Å in the MD box of DPQAO‐F, leaving little chance for strong intermolecular interactions. Specifically, three pairs of guest and host molecules with the closest distances (Figure 4c) were extracted from their boxes. QAO‐mCP pairs show significantly closer intermolecular distances in the 3.0–4.7 Å range, where guest and host molecules are prone to adapt face‐to‐face packing. Conversely, a longer intermolecular distance of 3.5–6.2 Å was found in DPQAO‐F‐mCP pairs with most π planes (Cz, PF, or QAO) tilted or staggered to each other.^[^ ^22^, ^32^ ^]^ These results illustrate much weaker intermolecular interactions in DPQAO‐F:mCP blends.

### 2.5 Device Performance

To evaluate the EL performance of these novel carbonyl/nitrogen‐based MR‐TADF emitters, OLEDs were fabricated with an architecture of ITO/TAPC (40 nm)/TCTA (10 nm)/mCP (10 nm)/EML (20 nm)/TmPyPB (40 nm)/LiF (1 nm)/Al (100 nm). EML is the emissive layer with 1, 2, 4, or 8 wt.% of DPQAO‐F/DPQAO‐M doped in mCP. The device stack and chemical structures of the materials employed in devices are shown in Figure S15 (Supporting Information).

**Figure 5:**
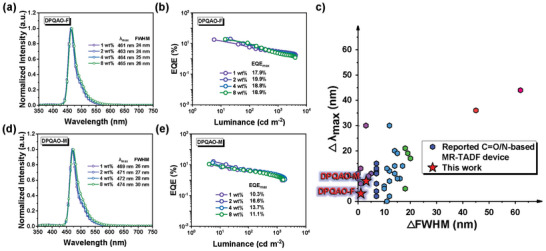
EL spectrum and external quantum efficiency versus luminance curses for DPQAO‐M (a–b) and DPQAO‐F (d–e) in doping ratios of 1–8 wt.%; c) Comparison of emission profile change for reported blue C═O/N based MR‐emitter (DPQAO‐M and DPQAO‐F are also presented as the red star for study).^[^ ^23^, ^26^, ^30^, ^33^, ^34^, ^35^, ^36^, ^37^, ^38^, ^39^, ^40^, ^41^, ^42^, ^43^, ^44^, ^45^, ^46^, ^47^ ^]^

The device performance is summarized in **Figure** **5** and the key data is listed in **Table** **1**. DPQAO‐F‐based devices present lower turn‐on voltages than those of DPQAO‐M devices (3.5–3.2 vs 4.1–3.5 V), implying PF does not undermine the electrical properties or hinder the energy transferring process in EML. As illustrated in **Figure** **5**a,b, EL spectra with peaks ≈461–463 nm were recorded for DPQAO‐F‐based devices with small FWHMs of 24–26 nm upon increasing doping ratio from 1–8 wt.%, consisting of the solution and thin‐film state PL properties. 2 wt.% DPQAO‐F doped device shows the best performance with EQE_max_ of 19.9% with pure‐blue CIE coordinates of (0.134, 0.118) and small EQE_max_ variation was observed as the doping concentration increased (18.8% and 18.9% for EML with 4 and 8 wt.%, respectively). Compared to the reported QAO‐based MR‐emitter,^[^ ^23^ ^]^ DPQAO‐F shows the strongest persistency against redshifting (∆*λ*_max_) and profile broadening (∆FWHM) from solution to device (Figure 5c). In sharp contrast, DPQAO‐M‐based devices exhibited serious EQE_max_ dropping (10.2%→16.6%→13.7%→11.1% of 1, 2, 4, and 8 wt.% doped devices, respectively) accompanied by broadening of EL profile (FWHM from 26–30 nm), resulting in the increased CIE_y_ from 0.187 to 0.230 (Figure 5d,e). Consisting with thin‐film PL properties results, such a small ΔFWHM and ΔEQE in DPQAO‐F‐based devices verify the success of the molecular design and the effectiveness of PF group in suppressing intermolecular interaction (guest‐host and guest‐guest) in an external electric field.

**Table 1:** Device performances of QAO‐, DPQAO‐F‐ and DPQAO‐M‐based TADF‐OLEDs.

Emitters	Doping ratio [%]	V_on_ ^a)^ [V]	*λ*_EL_ ^b)^ [nm]	FWHM ^c)^ [nm]	η_c_ ^d)^ [cd A^−1^]	η_p_ ^e)^ [lm W^−1^]	η_ext_ ^f)^ [%]	CIE ^g)^ (x, y)
DPQAO‐M	1	4.1	469	26	12.3, 5.3, 2.5	9.4, 3.1, 0.9	10.2, 4.1, 1.9	0.129, 0.187
	2	3.8	471	27	18.1, 6.2, 2.9	15.0, 4.0, 1.2	16.6, 4.6, 2.2	0.126, 0.198
	4	3.8	472	28	18.8, 6.2, 3.1	15.5, 3.9, 1.3	13.7, 4.4, 2.2	0.127, 0.209
	8	3.5	474	30	15.5, 6.1, 2.6	13.9, 4.2, 1.1	11.1, 4.1, 1.8	0.126, 0.230
DPQAO‐F	1	3.5	461	24	17.1, 5.7, 2.7	15.4, 4.4, 1.4	17.9, 5.7, 2.8	0.136, 0.111
	2	3.5	463	24	22.4, 8.8, 3.0	20.1, 7.0, 1.6	19.9, 8.3, 3.0	0.134, 0.118
	4	3.5	464	25	23.2, 10.6, 2.8	20.8, 8.6, 1.5	18.8, 9.4, 2.6	0.134, 0.132
	8	3.2	465	26	22.4, 12.8, 2.8	22.0, 11.3, 1.5	18.9, 10.1, 2.3	0.134, 0.150

^a)^ Turn‐on voltage;

^b)^ EL emission maximum at 1000 cd m^−2^;

^c)^ Full width at half‐maximum of the EL spectrum;

^d)^ Maximum current efficiency and current efficiency at luminances of 100 and 1000 cd m^−2^;

^e)^ Maximum power efficiency and power efficiency at luminances of 100 and 1000 cd m^−2^;

^f)^ Maximum external EL quantum efficiency and external EL quantum efficiency at luminances of 100 and 1000 cd m^−2^;

^g)^ Commission Internationale de l’Éclairage (CIE) chromaticity coordinate.

**Figure 6:**
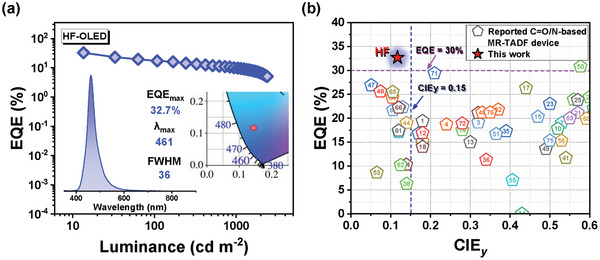
a) EQE versus luminance curses of hyperfluorescent‐device (inset: EL spectra and CIE 1931 chromaticity diagram). b) Comparison of EQEs for reported representative devices based on N/C═O MR system. The performance of deep‐blue HF devices based on DPQAO‐F fabricated in this study (red stars) is also compared.^[^ ^23^, ^25^, ^26^, ^30^, ^33^, ^34^, ^35^, ^36^, ^37^, ^38^, ^39^, ^40^, ^41^, ^42^, ^43^, ^44^, ^45^, ^46^, ^47^, ^48^, ^49^, ^50^, ^51^, ^52^, ^53^, ^54^, ^55^ ^]^

To further characterize the electroluminescent performance of DPQAO‐F, hyperfluorescent (HF)‐OLED was fabricated using DPQAO‐F as the terminal emitter (ITO/TAPC (50 nm)/TCTA (10 nm)/mCP (10 nm)/PPF:30 wt.% TDBA‐SPX:1.5 wt.% emitters (25 nm)/PPF (5 nm)/3TPYMB (50 nm)/LiF (1 nm)/Al (100 nm)). To compensate for the slow RISC of DPQAO‐F, TDBA‐SPX^[^ ^31^ ^]^ was chosen as a sensitizer because of its high triplet energy (2.93 eV) and fast RISC rate (1.53 × 10^6^ s^−1^) and significant overlap between its PL spectra and the absorption of DPQAO‐F (Figure S17, Supporting Information). As depicted in **Figure** **6** and summarized in **Table** **2**, the hyper‐device presents a narrow deep‐blue EL emission peaking at 461 nm with small FWHMs of 36 nm, leading to a fantabulous CIE coordinate of (0.146, 0.117). Furthermore, impressive EL performances with a high EQE_max_ of 32.7% and PE_max_ of 28.0 lm W^−1^ were recorded, manifesting the best device performance among nitrogen/carbonyl MR‐TADF emitters in the deep‐blue region. Angle‐dependent photoluminescence measurement was carried out to understand the origin of the high EQE better. The horizontal transit dipole orientation ratio of the thin film of the HF emissive layer was 82% (Figure S22a, Supporting Information), which contributed to the lift of the upper limit and EQE_max_ theoretically. Among previously reported nitrogen/carbonyl‐based MR‐TADF OLEDs (Figure 6b), this work is the first example that simultaneously achieves deep‐blue emission and EQE over 30%.

**Table 2:** Device performances of DPQAO‐F‐based HF‐OLED.

Emitter	Doping ratio [%]	V_on_ ^a)^ [V]	*λ*_EL_ ^b)^ [nm]	FWHM ^c)^ [nm]	η_c_ ^d)^ [cd A^−1^]	η_p_ ^e)^ [lm W^−1^]	η_ext_ ^f)^ [%]	CIE ^g)^ (x, y)
DPQAO‐F	1.5	3.6	461	36	32.03, 19.2, 9.5	28.0, 13.5, 4.0	32.7, 17.6, 9.9	0.146, 0.117

^a)^ Turn‐on voltage;

^b)^ EL emission maximum at 6 V;

^c)^ Full width at half‐maximum of the EL spectrum;

^d)^ Maximum current efficiency and current efficiency at luminances of 100 and 1000 cd m^−2^;

^e)^ Maximum power efficiency and power efficiency at luminances of 100 and 1000 cd m^−2^;

^f)^ Maximum external EL quantum efficiency and external EL quantum efficiency at luminances of 100 and 1000 cd m^−2^;

^g)^ Commission Internationale de l’Éclairage (CIE) chromaticity coordinate.

## 3 Conclusion

In summary, we demonstrate stepwise engineering C═O/N‐based (QAO) MR‐TADF emitters to achieve narrowband deep‐blue emission in the solid state and devices. By locking the QAO resonant core, the narrower PL emission in solution was realized in two new compounds, DPQAO‐M and DPQAO‐F. With PF as a shielding group, DPQAO‐F delivered superior PL performance (*Φ*_PL_ up to 84.2%) and stable color purity (∆FWHM ≤ 3 nm) in the solid state. Single crystal analysis and MD simulations reveal that QAO and DPQAO‐M bear strong intermolecular interactions from both host and guest molecules, presenting strong media/concentration‐dependent PL properties. At the same time, DPQAO‐F has the largest intermolecular distance with well‐suppressed host‐guest and guest‐guest interactions. Consequently, deep‐blue OLEDs based on DPQAO‐F exhibit stable performance upon a doping range of (1–8 wt.%) with emission peaks at 461–465 nm, EQE_max_ of 17.9–19.9% and ultranarrow FWHM of 24–26 nm. We believe that the performance of *L*‐QAO's TADF device can be further improved by rationally modifying the molecular structure to enhance its S_1_‐S_0_ transition oscillator, increase its horizontal alignment, or narrow down its Δ*E*_ST_. Furthermore, HF‐OLED based on DPQAO‐F exhibits deep‐blue emission peaking at 461 nm and record‐high EQE_max_ at 32.7% with a small CIE_y_ of 0.117. These results show PF as an ideal inert shielding group for deep‐blue MR emitters. We believe the example presented in this work will inspire molecular design in various MR‐TADF emitters to achieve accordance in design and applications.

## Conflict of Interest

The authors declare no conflict of interest.

## Supporting information

Supporting Information

## Data Availability

The data that support the findings of this study are available from the corresponding author upon reasonable request.
