# Supplementary material for: Stepwise Toward Pure Blue Organic Light‐Emitting Diodes by Synergetically Locking and Shielding Carbonyl/Nitrogen‐Based MR‐TADF Emitters
Source: Adv Sci (Weinh). 2024 May 5;11(28):2401664. doi: 10.1002/advs.202401664 (PMC11267287; doi:10.1002/advs.202401664)
Supplement: Supplementary file 1 — Supporting Information [file ADVS-11-2401664-s001.docx]

Supporting Information

**Stepwise Towards Pure Blue Organic Light-Emitting Diodes by Synergetically Locking and Shielding Carbonyl/Nitrogen-Based MR-TADF Emitters**

Jie-Rong Yu^‡^, Hong-Ji Tan^‡^, Xiu-Qi Gao, Bing Wang, Zhi-Qiang Long, Jia-Li Liu, Zhi-Zhong Lin, Xing-Yi Li, Ze-Lin Zhu*, Jing-Xin Jian, Qing-Xiao Tong* and Chun-Sing Lee*

**General information**

The nuclear magnetic resonance (NMR) spectra were recorded on the Bruker Ascend^TM^ 400 MHz NMR spectrometer with tetramethylsilane (TMS) as an internal standard. Decomposition temperature (*T_d_*, at 5 wt% lost) is recorded with Shimadzu TA50 at a heating rate of 10 °C min^-1^ under the nitrogen atmosphere. Glass transition temperature (*T_g_*) is determined on a Shimadzu DSC60. High-resolution mass spectra were recorded using a 4800 Plus MALDI TOF/TOF™ analyzer. Absorption and photoluminescence spectra are measured with a Shimadzu UV2600 UV-Vis spectrophotometer and a Hitachi F7000 Luminescence spectrophotometer, respectively. Cyclic voltammetry is performed on a CHI600E electrochemical analyzer with a three-electrode system (a glassy carbon electrode as the working electrode, a platinum wire as the auxiliary electrode, an Ag/AgCl electrode as the pseudo-reference electrode with Fc/Fc+ as external standard. The HOMO energy levels of the compounds were calculated according to the formula: *E*_HOMO_ (eV) = -[4.8 + (*E*_1/2(ox/red)_ - *E*_1/2(Fc+/Fc)_)] eV. The LUMO energy levels of the compounds were then deduced from the HOMO levels and the UV-Vis absorption on-sets of the longer wavelength. Nitrogen saturated dichloromethane with 0.1 M tetrabutylammonium hexafluorophosphate (supporting electrolyte) is used as the solvent. The devices were fabricated on pre-cleaned 50 nm ITO-coated glass substrates. Before use, the substrates were swabbed with Decon-90 solution and rinsed with deionized water. The solvent on the surface was removed with dry N_2_ flow, and the clean substrates were then stored in an oven at 100 °C. After a 30-min UV-ozone treatment, the substrates were transferred into a deposition chamber. All the organic films were deposited at a rate of 0.5-1.0 Å s^-1^ by thermal evaporation in a deposition chamber with a base vacuum of 5 × 10^-6^ Torr. The *J*-*V* (current density-voltage) characteristics were recorded using a Keithley 2400 Sourcemeter. EL spectra/radiation and Commission Internationale de l’Éclairage (CIE) color coordinates were measured using a PMA-12 photonic multichannel analyzer (Hamamatsu). Device measurements were performed under ambient conditions without encapsulation. Angle-dependent photoluminescence (PL) was conducted to measure the horizontal dipole ratio (HDR, Θ//) of the light emission molecules in thin films.^[1]^ P-polarized angle-dependent light emissions of thin films were measured by Fluxim. The refractive index of the materials was measured by MEL broadband spectroscopic Mueller matrix ellipsometer or extracted by the Setfos database.

**Computation method**

The density functional theory (DFT) and time-dependent DFT (TD-DFT) calculations were both carried out at b3lyp/6–31G(d, p) levels using the Gaussian 09 program package.^[2]^ Further quantum chemistry analysis was performed using a multifunctional wavefunction analyzer (Multiwfn 3.7) and visualized with the VMD program (1.9.3).^[3]^ The Huang-Rhys (HR) factors and reorganization energy (λ) were predicted using the Molecular Materials Property Prediction Package (MOMAP) software.^[4]^

**Molecular dynamic (MD) simulations details**

All MD simulations were performed with the GROMACS package using the general amber force field (GAFF) force field and restricted electrostatic potential (RESP) atomic charge.^[5,6]^ Two 10 × 10 × 10 nm cells composing random distribution of mCP: QAO/DPQAO-F host-guest system with a molar ratio of 294:6 were initially prepared by Packmol software, respectively.^[7]^ The conjugated gradient method was used for energy minimization to eliminate the apparent irrational repulsion in the system. A 100-ps equilibrium MD simulation was conducted under the NVT ensemble to make the system fully balanced. Then 5-ns MD simulation using NPT ensemble at 1 atm and 298.15 K was first performed for equilibration. Finally, the production MD simulations were carried out for 10 ns. At the NVT ensemble, the Berendsen was used to keep the temperature constant. The velocity-rescale temperature coupling with a time constant of 0.2 ps and Berendsen pressure coupling with a time constant of 0.5 ps were used for the NPT ensemble. The periodic boundary conditions were used in the MD process. The long-range electrostatic interactions were corrected by the particle mesh Ewald (PME) algorithm.^[8]^ A cutoff radius of 10 Å was applied to truncate the van der Waals forces.

**Single crystal culture and analysis**

Single crystals of DPQAO-F (CCDC 2294093), DPQAO-M (CCDC 2294094), and QAO (CCDC 2296491) were obtained by slow diffusion of acetonitrile into their saturated dichloromethane solutions, and their X-ray diffraction measurement was performed on a single crystal X-ray diffractometer (Oxford Gemini S Ultra) with Cu Kα radiation (λ = 1.54178 Å). These data can be obtained free of charge from The Cambridge Crystallographic Data Centre via www.ccdc.cam.ac.uk/data_request/cif.

**Materials and synthesis**

**Scheme S1**. Reported molecules are mentioned in this work.

**Scheme S2.** Synthetic routes and chemical structure of 12,12-diphenyl-2,10-bis(9-phenyl-9H-fluoren-9-yl)-4H-benzo[9,1]quinolizino[3,4,5,6,7-defg]acridine-4,8(12H)-dione (DPQAO-F) and 2,10-dimethyl-12,12-diphenyl-4H-benzo[9,1]quinolizino[3,4,5,6,7-defg]acridine-4,8(12H)-dione (DPQAO-M)

Commercially available reagents and solvents were purchased and used without further purification unless otherwise stated. Compounds **1** were synthesized by following previous reports.^[9]^ Compound QAO (quinolino[3,2,1-de]acridine-5,9-dione) was synthesized by following previous reports. ^[10]^

***Synthesis of dimethyl 2-(9,9-diphenylacridin-10(9H)-yl)isophthalate (3):***

Compound dimethyl 2-bromoisophthalate (2.73 g, 10.00 mmol), 9,9-diphenyl-9,10-dihydroacridine (3.33 g, 10.00 mmol), potassium carbonate (2.07 g, 15.00 mmol), copper (I) iodide (190.45 mg, 1.00 mmol), 2,2,6,6-tetramethylheptane-3,5-dione (184.28 mg, 1.00 mmol) and activated copper powder (63.55 mg, 1.00 mmol) were combined with 150 mL 1-butoxybutane in a round bottom flask equipped with a reflux condenser and magnetic stir bar. The reaction mixture was heated to 160 °C for 48 h under argon. After cooled to room temperature, the reaction mixture was filtered, the solvent was removed under vacuum condition, and then the residue was purified by column chromatography on silica gel using petroleum ether/dichloromethane (1/4, v/v) as eluent, yellow solid was finally obtained. (3.26 g, yield: 62%) ^1^H NMR (400 MHz, DMSO-*d6*) δ 8.17 (d, *J* = 7.7 Hz, 2H), 7.82 (t, *J* = 7.8 Hz, 1H), 7.28 (t, *J* = 7.5 Hz, 4H), 7.20 (t, *J* = 7.2 Hz, 2H), 6.99 – 6.94 (m, 6H), 6.82 – 6.78 (m, 2H), 6.75 (dd, *J* = 7.8, 1.7 Hz, 2H), 5.97 (d, *J* = 8.3 Hz, 2H), 3.04 (s, 6H). ^13^C NMR (101 MHz, DMSO-*d6*) δ 165.60, 148.43, 141.32, 137.34, 135.12, 134.62, 131.15, 130.21, 128.16, 127.38, 126.91, 126.57, 120.36, 113.50, 55.98, 52.26.

***Synthesis of*** ***dimethyl 2-(2,7-dimethyl-9,9-diphenylacridin-10(9H)-yl)isophthalate (9)：***

Compound 9 was synthesized according to the same procedure as for compound 3, yellow powder was finally obtained. (3.64 g, yield: 64.98 %) ^1^H NMR (400 MHz, Methylene Chloride-*d*_2_) δ 8.18 (d, *J* = 7.8 Hz, 2H), 7.71 (t, *J* = 7.8 Hz, 1H), 7.31 (dd, *J* = 8.2, 6.6 Hz, 4H), 7.27 – 7.23 (m, 2H), 7.12 – 7.08 (m, 4H), 6.83 (dd, *J* = 8.4, 2.0 Hz, 2H), 6.69 (d, *J* = 1.9 Hz, 2H), 6.00 (d, *J* = 8.4 Hz, 2H), 3.18 (s, 6H), 2.20 (s, 6H). ^13^C NMR (101 MHz, Methylene Chloride-*d*_2_) δ 165.56, 148.46, 139.57, 138.62, 134.81, 134.72, 131.60, 130.34, 128.78, 128.70, 127.56, 127.49, 126.75, 125.96, 112.95, 56.12, 51.87, 20.42.

***Synthesis of dimethyl 2-(9,9-diphenyl-2,7-bis(9-phenyl-9H-fluoren-9-yl)acridin-10(9H)-yl)isophthalate (5)：***

Compound 5 was synthesized according to the previous report.^[11]^ In a 250 mL three-necked round-bottomed flask charged with compound 1 (1.80 g, 3.42 mmol) and BF_3_·Et_2_O (2.1 mL) in 80 mL of dry dichloromethane, a solution of PFOH (500 mg 1.94 mmol) in 60 mL dry dichloromethane was added dropwise. Then the reaction mixture was stirred at room temperature for 4 h. Water (100 mL) was added to quench the reaction. The mixture was separated, and the aqueous phase was extracted with dichloromethane for 3 times. The combined organic layers were washed with brine and dried over MgSO_4_. After removal of the solvent, the crude product was purified by column chromatography using an eluent of dichloromethane: petroleum ether (1:10) to afford light yellow product solid (1.07 g, 73%). ^1^H NMR (400 MHz, Methylene Chloride-*d*_2_) δ 8.11 (d, *J* = 7.7 Hz, 2H), 7.77 (d, *J* = 7.5 Hz, 4H), 7.62 (t, *J* = 7.8 Hz, 1H), 7.36 (td, *J* = 7.4, 1.2 Hz, 4H), 7.23 (td, *J* = 7.3, 1.4 Hz, 10H), 7.18 – 7.15 (m, 10H), 7.05 (dd, *J* = 5.4, 2.3 Hz, 6H), 7.00 – 6.96 (m, 4H), 6.62 (dd, *J* = 8.6, 2.3 Hz, 2H), 5.88 (d, *J* = 8.6 Hz, 2H), 3.14 (s, 6H). ^13^C NMR (101 MHz, Methylene Chloride-*d*_2_) δ 165.50, 151.40, 148.46, 146.07, 140.03, 139.97, 138.21, 136.52, 134.91, 134.42, 132.72, 130.12, 128.84, 127.99, 127.77, 127.48, 127.44, 127.27, 127.13, 126.34, 125.90, 125.47, 120.03, 112.55, 64.67, 56.29, 51.84.

***Synthesis of 2-(9,9-diphenyl-2,7-bis(9-phenyl-9H-fluoren-9-yl)acridin-10(9H)-yl)isophthalic acid (******6)：***

Compound 5 (3.6 g, 3.58 mmol) and sodium hydroxide (1.43 g, 35.8 mmol) in a solution of 1:1 ethanol/water (150 mL) heated to reflux for 12 h. Acidification with concentrated hydrochloric acid precipitated the triarylamine diacid, which was collected by vacuum filtration and oven-dried (80 °C) overnight, then employed directly without further purification. Yield: 3.26 g (93.2%)

***Synthesis of 2-(2,7-dimethyl-9,9-diphenylacridin-10(9H)-yl)isophthalic acid (10)：***

Compound 9 (3.4 g, 6.2 mmol) and sodium hydroxide (2.46 g, 61.4 mmol) in a solution of 1:1 ethanol/water (150 mL) heated to reflux for 12 h. Acidification with concentrated hydrochloric acid precipitated the triarylamine diacid, which was collected by vacuum filtration and oven-dried (80 °C) overnight, then employed directly without further purification. Yield: 3.10 g (96.1%)

***Synthesis of*** ***12,12-diphenyl-2,10-bis(9-phenyl-9H-fluoren-9-yl)-4H-benzo[9,1]quinolizino[3,4,5,6,7-defg]acridine-4,8(12H)-dione (DPQAO-F):***

Compound 6 (3.0 g, 3.07 mmol) was dispersed in dry dichloromethane (100 mL) in a three-neck round-bottom flask equipped with a magnetic stir bar and reflux condenser with a drying tube. Two drops of N, N-dimethylformamide were added followed by oxalyl chloride (3.22 mL, 6.45 mmol). The reaction was heated to reflux for 0.5 h. Sn (IV) chloride (0.75 mL, 6.45 mmol) was added and the reaction refluxed for an additional 3 h. The reaction mixture was added dropwise to an aqueous solution of sodium hydroxide and extracted with dichloromethane. The organic layer is dried over sodium sulfate and concentrated. The crude product was then purified by flash chromatography using DCM/petroleum ether (3/1, v/v) as the eluent to give a pure yellow solid. Yield: 1.51 g (52.3%). This compound was further purified by sublimation before being used in device fabrication. ^1^H NMR (400 MHz, Methylene Chloride-*d*_2_) δ 8.85 (dd, *J* = 7.7, 2.3 Hz, 2H), 8.16 (d, *J* = 2.5 Hz, 2H), 7.82 (d, *J* = 7.6 Hz, 4H), 7.69 – 7.64 (m, 1H), 7.56 (d, *J* = 2.5 Hz, 2H), 7.44 – 7.40 (m, 4H), 7.31 – 7.27 (m, 4H), 7.21 (dtd, *J* = 9.1, 6.0, 5.2, 2.6 Hz, 16H), 7.09 – 7.06 (m, 4H), 6.81 (dt, *J* = 6.8, 1.6 Hz, 4H). ^13^C NMR (101 MHz, Methylene Chloride-*d*_2_) δ 176.78, 150.19, 146.43, 144.79, 142.64, 140.11, 138.22, 137.27, 133.79, 131.87, 129.69, 129.59, 128.54, 128.38, 128.22, 128.19, 128.03, 127.88, 127.85, 127.73, 126.90, 126.89, 125.76, 124.44, 123.85, 122.71, 122.04, 120.43, 64.74, 55.73. MALDI-TOF MS (mass m/z): 942.6130 [M]^+^. Calcd for C_71_H_43_NO_2_: 942.1300.

***Synthesis of*** ***2,10-dimethyl-12,12-diphenyl-4H-benzo[9,1]quinolizino[3,4,5,6,7-defg]acridine-4,8(12H)-dione (DPQAO-M):***

Compound 10 (3.1 g, 5.9 mmol) was dispersed in dry dichloromethane (100 mL) in a three-neck round-bottom flask equipped with a magnetic stir bar and reflux condenser with a drying tube. Two drops of N, N-dimethylformamide were added followed by oxalyl chloride (6.2 mL, 12.39 mmol). The reaction was heated to reflux for 0.5 h. Sn (IV) chloride (1.45 mL, 12.39 mmol) was added and the reaction refluxed for an additional 3 h. The reaction mixture was added dropwise to an aqueous solution of sodium hydroxide and extracted with dichloromethane. The organic layer is dried over sodium sulfate and concentrated. The crude product was then purified by flash chromatography using DCM/petroleum ether (3/1, v/v) as the eluent to give a bright yellow solid. Yield: 1.90 g (65.6%). This compound was further purified by sublimation before being used in device fabrication. ^1^H NMR (400 MHz, Methylene Chloride-*d*_2_) δ 8.85 (dd, *J* = 7.7, 2.0 Hz, 2H), 8.32 (d, *J* = 2.4 Hz, 2H), 7.64 (td, *J* = 7.7, 2.0 Hz, 1H), 7.30 – 7.25 (m, 8H), 6.98 – 6.94 (m, 4H), 2.45 (s, 6H). ^13^C NMR (101 MHz, Methylene Chloride-*d*_2_) δ 177.02, 146.05, 137.54, 137.27, 134.70, 133.63, 133.42, 131.71, 129.99, 128.12, 127.00, 126.38, 123.42, 122.70, 122.31, 55.83, 20.70. MALDI-TOF MS (mass m/z): 490.3504 [M]^+^. Calcd for C_35_H_23_NO_2_: 489.5740.

**Figure S1**. Calculated molecular electrostatic potential (ESP) and molecular polarity index (MPI) of QAO, DPQAO-M, DPQAO-F.

**Figure S2**. Molecular design of carbonyl/nitrogen MR-system in this work.

**Figure S3**. Some examples of steric groups for commonly used and their xyz length.

**Figure S4**. Calculated natural transition orbitals of S_1_-S_0_ and T_1_-S_0_ transition of QAO, DPQAO-M, and DPQAO-F in B3LYP/6-31G(d,p) level under gas phase.

**Figure S5.** Low-temperature fluorescence (LTFL) and low-temperature phosphorescence (LTPh) spectra of a) DPQAO-M and b) DPQAO-F in degassed toluene. c) LTPh spectra of diphenylfluorene (PF). d) Transient PL decay of DPQAO-M and DPQAO-F in degassed toluene.

**Figure S6.** Photoluminescent spectra of QAO^[10]^, DPQAO-M, and DPQAO-F in dilute toluene solution

**Figure S7.** Solvatochromism PL spectra of a) DPQAO-M and b) DPQAO-F in n-hexane, toluene, tetrahydrofuran (THF), dichloromethane (DCM) and dimethylsulfoxide (DMSO).

**Figure S8.** Steady-state PL spectrum of 4 wt% QAO, DPQAO-M, and DPQAO-F doped in different hosts (CzSi, mCP, TPSO1, and DPEPO), respectively.

**Figure S9.** Photoluminescence quantum yield (PLQY) of DPQAO-M doped in 1, 2, 4, 8, and 15 wt% mCP film.

**Figure S10.** Low-temperature fluorescence (LTFL), low-temperature phosphorescence (LTPh) spectra, and temperature-dependent transient PL decay spectra (77-350K) of a), c) DPQAO-M and b), d) DPQAO-F in 2 wt% doped mCP films.

**Figure S11.** a) Thermal gravimetric analysis and b) differential scanning calorimetry thermograms of curves

**Figure S12.** Cyclic voltammograms of a) DPQAO-M and DPQAO-F b) ferrocene in dichloromethane.

**Figure S13.** Energy alignment and proposed interaction for DPQAO-M, DPQAO-F, and mCP. Orbital energy alignment of mCP and a) DPQAO-M; c) DPQAO-F. The blue dashed lines represent the equalized energy level for the LUMO and SOMO. Schematic of the state energy alignment for mCP and b) DPQAO-M ; d) DPQAO-F.

**Figure S14**. Structural drawing of QAO, DPQAO-M, DPQAO-F in crystal.

**Figure S15.** Configurations of TADF OLEDs and chemical structures of materials used.

**Figure S16**. Current density–voltage–luminance (J–V–L) curses and current efficiency (CE) and power efficiency (PE) versus luminance curses for TADF-OLED of DPQAO-F(a-b) and (c-d).

**Figure S17**. a) The absorption spectrum of DPQAO-F and emission spectra of TDBA-SPX measured in diluted toluene. b) emission spectra of the mixed films of PPF: 30 wt% TDBA-SPX; PPF: 1.5 wt% DPQAO-F and PPF: 30 wt% TDBA-SPX: 1.5 wt% DPQAO-F. c) Transient PL decay of PPF: 30 wt% TDBA-SPX and PPF: 30 wt% TDBA-SPX: 1.5 wt% DPQAO-F.

**Figure S18**. Configurations of hyperfluorescent (HF) OLED and chemical structures of materials used.

**Figure S19**. a) Current density–voltage–luminance (J–V–L) curses and b) external quantum efficiency (EQE), current efficiency (CE), and power efficiency (PE) versus luminance curses for HF-OLED.

**Figure S20**. Energy transfer process of HF device.

**Figure S21**. a) Current density–voltage–luminance (J–V–L) curses and b) external quantum efficiency (EQE), current efficiency (CE), and power efficiency (PE) versus luminance curses for TDBA-SPX based TADF-OLED.

**Figure S22**. The angle-dependent photoluminescent intensity of p-polarized light from the thin film of the a) HF emissive layer (blue) and b) 30 wt% TDBA-SPX: PPF.

**Table S1.** Photophysical data of the DPQAO-M and DPQAO-F in solution

| Emitters | λ_abs_^[a]^  [nm] | λ_em_^[b]^  [nm] | FWHM^[c]^  [nm] | Δλ^[d]^  [nm] | E_g_^[e]^  [eV] | HOMO^[f]^  [eV] | LUMO^[f]^  [eV] | CIE^[g]^  (x, y) | S_1_^[h]^  [eV] | T_1_^[i]^  [eV] | ∆E_ST_ ^[j]^  [eV] | *Φ*_PL_^[k]^  [%] | τ_p_^[l]^  [ns] |
| --- | --- | --- | --- | --- | --- | --- | --- | --- | --- | --- | --- | --- | --- |
| DPQAO-M | 447 | 461 | 24 | 14 | 2.68 | -5.98 | -3.30 | (0.14, 0.19) | 2.67 | 2.42 | 0.25 | 32.3 | 5.38 |
| DPQAO-F | 445 | 458 | 23 | 14 | 2.70 | -6.03 | -3.33 | (0.14, 0.11) | 2.68 | 2.44 | 0.24 | 34.1 | 4.86 |

[a] Absorption peak at room temperature measured in toluene solution (1.0×10^-5^ M); [b] Emission peak at room temperature measured in toluene solution (1.0×10^-5^ M); [c] Full width at half maximum of PL spectrum; [d] Stokes shift, Δλ = λ_em_ – λ_abs_; [e] Optical energy gap estimated from the absorption onset; [f] HOMO level measured from the oxidation potential in 10^-3^ M dichloromethane solution by cyclic voltammetry with ferrocene as the external standard and LUMO level calculated from HOMO+E_g_; [g] Commission Internationale de l'Éclairage (CIE) chromaticity coordinates measured in toluene solution; [h] Estimated from the onsets of the low-temperature fluorescence spectrum at 77 K; [i] Estimated from the onsets of the low-temperature phosphorescence spectrum at 77 K; [j] Estimated from S_1_ and T_1_; [k] Photoluminescence quantum yield measured in toluene solution; [l] Prompt decay fluorescence lifetime in toluene solution.

**Table S2.** Photophysical properties and kinetic parameters of the DPQAO-M and DPQAO-F in doped mCP films

| Emitters | S_1_^[c]^  [eV] | T_1_^[d]^  [eV] | ∆E_ST_^[e]^  [eV] | Doping ratio  [wt%] | | λ_PL_^[a]^  [nm] | FWHM^[b]^  [nm] | | *Φ*_PL_^[f]^  [%] | *Φ*_PF_^[g]^  [%] | *Φ*_DF_^[h]^  [%] | τ_p_^[i]^  [ns] | τ_d_^[j]^  [μs] | *k*_p_^[k]^  [10^8^ S^-1^] | *k*_d_^[l]^  [10^3^ S^-1^] | *k*_r_^S[m]^  [10^7^ S^-1^] | *k*_ISC_^[n]^  [10^7^ S^-1^] | *k*_RISC_^[o]^  [10^4^ S^-1^] | *k*_nr_^S [p]^  [10^6^ S^-1^] |
| --- | --- | --- | --- | --- | --- | --- | --- | --- | --- | --- | --- | --- | --- | --- | --- | --- | --- | --- | --- |
| DPQAO-M | 2.61 | 2.39 | 0.22 | 1 | 470 | | | 31 | 44.9 | 9.9 | 35.0 | 4.67 | 189.96 | 2.14 | 5.26 | 2.11 | 4.70 | 2.40 | 25.90 |
|  |  |  |  | 2 | 472 | | | 32 | 77.7 | 18.2 | 59.5 | 4.16 | 163.64 | 2.40 | 6.11 | 4.38 | 5.64 | 2.60 | 12.58 |
|  |  |  |  | 4 | 473 | | | 34 | 65.6 | 13.7 | 51.9 | 3.83 | 151.59 | 2.61 | 6.60 | 3.58 | 5.46 | 3.16 | 18.77 |
|  |  |  |  | 8 | 474 | | | 37 | 56.9 | 13.2 | 43.7 | 2.46 | 133.21 | 4.07 | 7.51 | 5.36 | 9.41 | 3.24 | 40.58 |
| DPQAO-F | 2.66 | 2.45 | 0.21 | 1 | 460 | | | 23 | 67.7 | 21.6 | 46.1 | 3.97 | 122.33 | 2.52 | 8.17 | 5.43 | 8.02 | 2.57 | 25.91 |
|  |  |  |  | 2 | 461 | | | 24 | 84.2 | 16.0 | 68.2 | 3.37 | 125.44 | 2.97 | 7.97 | 4.76 | 5.66 | 4.18 | 8.94 |
|  |  |  |  | 4 | 461 | | | 25 | 80.5 | 13.6 | 66.9 | 3.13 | 108.27 | 3.19 | 9.24 | 4.36 | 5.41 | 5.45 | 10.56 |
|  |  |  |  | 8 | 462 | | | 26 | 77.9 | 18.9 | 59.0 | 2.70 | 107.54 | 3.70 | 9.30 | 7.00 | 8.98 | 3.83 | 19.86 |

[a] Emission peak at room temperature measured in the host-containing film; [b] Full width at half maximum of PL spectrum in the host-containing film; [c] Estimated from the onsets of the low-temperature fluorescence spectrum at 77 K; [d] Estimated from the onsets of the low-temperature phosphorescence spectrum at 77 K; [e] Estimated from S_1_ and T_1_; [f] Absolute photoluminescence quantum yield measured in mCP film; [g] Fractional quantum yield of prompt fluorescence emission; [h] Fractional quantum yield of delayed fluorescence emission; [i] Emission lifetime of prompt fluorescence; [j] Emission lifetime of delayed fluorescence; [k] Rate constant of prompt fluorescence; [l] Rate constant of delayed fluorescence; [m] Rate constant of fluorescence radiative decay; [n] Rate constant of intersystem crossing; [o] Rate constant of reverse intersystem crossing; [p] Rate constant of non-radiative decay.

In this work, the host-containing film of DPQAO-F and DPQAO-M do not exhibit phosphorescence emission at 300 K. In other words, the efficiency of phosphorescence is zero (*Ф*_Phos_ = 0). Thus, the quantum efficiency of delayed emission (*Ф*_DE_) is equal to the efficiency of delayed fluorescence (*Ф*_DF_). Thus, the quantum efficiencies of prompt (*Ф*_PF_) and delayed emission (*Ф*_DF_) are evaluated by the corrected estimation method and the rate constants were calculated according to the reported method.^[12]^

*k*_p_ = 1/*τ*_p_ (1)

*k*_d_ = 1/*τ*_d_ (2)

*k*_r_^S^ = *k*_p_*Φ*_PF_ (3)

*k*_nr_^S^ = *k*_p_*Φ*_PF_/*Φ*_PL_(1-*Φ*_PL_) (4)

*k*_ISC_ = *k*_p_*Φ*_DF_/*Φ*_PL_-*k*_d_*Φ*_DF_/*Φ*_PF_  (5)

*k*_RISC_ = *k*_d_*Φ*_PL_/*Φ*_PF_ (6)

where *k*_p_ and *k*_d_ are the radiative decay rate for prompt and delayed fluorescence, respectively; *Φ*_PL_ is the total photoluminescence quantum efficiency; *k*_r_^S^ and *k*_nr_^S^ are the radiative and non-radiative decay rate constants from a singlet excited state, respectively; *k*_ISC_ and *k*_RISC_ are the intersystem crossing and reverse intersystem crossing rate constants, respectively.

**Table S3.** Device performances of TDBA-SPX-based TADF-OLED.

| Emitter | V_on_ ^[a]^  [V] | *λ*_EL_^[b]^  [nm] | FWHM ^[c]^  [nm] | η_c_ ^[d]^  [cd A^-1^] | η_p_ ^[e]^  [lm W^-1^] | η_ext_ ^[f]^  [%] | CIE ^[g]^  (x, y) |
| --- | --- | --- | --- | --- | --- | --- | --- |
| TDBA-SPX | 3 | 453 | 52 | 20.0, 19.1, 16.2 | 20.9, 17.2, 8.8 | 24.8, 22.7, 18.8 | 0.146, 0.094 |

[a] Turn-on voltage; [b] EL emission maximum at 6V; [c] Full width at half-maximum of the EL spectrum; [d] Maximum current efficiency; [e] Maximum power efficiency; [f] Maximum external EL quantum efficiency; [g] Commission Internationale de l’Éclairage (CIE) chromaticity coordinate.

**Table S4.** Crystallographic data for QAO, DPQAO-M, and DPQAO-F.

| Compound | QAO | DPQAO-M | DPQAO-F |
| --- | --- | --- | --- |
| CCDC NO. | 2296491 | 2294094 | 2294093 |
| Empirical formula | C_20_H_11_NO_2_ | C_35_H_23_NO_2_ | C_71_H_43_NO_2_ |
| Formula weight | 297.30 | 489.54 | 1024.17 |
| Temperature/K | 233 | 299 | 218 |
| Crystal system | monoclinic | monoclinic | triclinic |
| Space group | P 21 | P 21/c | P-1 |
| a/Å | 3.8630(2) | 9.4770(1) | 12.021(6) |
| b/Å | 10.5046(4) | 19.7806(2) | 12.726(7) |
| c/Å | 16.1816(6) | 13.3869(1) | 18.233(10) |
| α/° | 90 | 90 | 72.118(19) |
| β/° | 95.161(2) | 101.944(1) | 84.528(17) |
| γ/° | 90 | 90 | 83.568(15) |
| Volume/Å^3^ | 653.98(5) | 2455.19(4) | 2632.3(20) |
| Z | 2 | 4 | 2 |
| ρ_calc_g/cm^3^ | 1.510 | 1.324 | 1.292 |
| μ/mm^‑1^ | 0.791 | 0.642 | 0.600 |
| F(000) | 308.0 | 1024.0 | 1072.0 |
| Reflections collected | 2461 | 4639 | 10596 |
| Independent reflections | 2243 | 4148 | 8654 |
| Data/restraints/parameters | 2461/1/208 | 4639/0/345 | 10596/0/723 |
| Goodness-of-fit on F^2^ | 1.125 | 1.060 | 1.066 |
| Final R indexes [I>=2σ (I)] | R_1_ = 0.0862,  wR_2_ = 0.2239 | R_1_ = 0.0441,  wR_2_ = 0.1191 | R_1_ = 0.0678,  wR_2_ = 0.1920 |
| Final R indexes [all data] | R_1_ = 0.0905,  wR_2_ = 0.2391 | R_1_ = 0.0476,  wR_2_ = 0.1222 | R_1_ = 0.0776,  wR_2_ = 0.2051 |

**Table S5.** Summarized key parameters of reported representative N/C=O MR emitters.

| Num. | Emitter | λ_PL_  （sol/film）  [nm] | FWHM_PL_  (sol/film)  [nm] | τ_DF_  (μs) | λ_EL_  [nm] | FWHM_EL_  [nm] | η_ext, max_  [%] | CIE (x, y） | Reference |
| --- | --- | --- | --- | --- | --- | --- | --- | --- | --- |
| 1 | QAO | 466/- | 32/- | 93.3 | 468 | 39 | 19.4 | (0.13, 0.18) | [10] |
| 2 | QAO-Dad | 548/- | - | 7.8 | 552 | - | 23.9 | (0.41, 0.56) | [10] |
| 3 | 3-PhQAD | 466/478 | 30/55 | 250 | 480 | 44 | 19.1 | (0.13, 0.32) | [13] |
| 4 | 7-PhQAD | 464/472 | 22/58 | 474 | 472 | 34 | 18.7 | (0.12, 0.24) | [13] |
| 5 | DiKTa | 453/463 | 27/37 | 15 | 465 | 39 | 14.7 | (0.14, 0.18) | [14] |
| 6 | Mes3DiKTa | 468/477 | 29/37 | 20 | 480 | 36 | 21.1 | (0.12, 0.32) | [14] |
| 7 | DDiKTa | 470/490 | 47/61 | 1.17 | 500 | 59 | 19.0 | (0.18, 0.53) | [15] |
| 8 | DQAO | 465/472 | 33/34 | 110.6 | 472 | 34 | 15.2 | (0.12, 0.18) | [16] |
| 9 | OQAO | 520/534 | 36/45 | 204.7 | 532 | 45 | 20.3 | (0.32, 0.65) | [16] |
| 10 | SQAO | 522/560 | 54/60 | 78.4 | 564 | 72 | 17.8 | (0.47, 0.52) | [16] |
| 11 | QA-PF | 465/478 | 23/30 | 346.9 | 474 | 27 | 16.8 | (0.12, 0.17) | [17] |
| 12 | QA-PCN | 462/477 | 25/34 | 223.9 | 473 | 30 | 16.9 | (0.12, 0.18) | [17] |
| 13 | QA-PMO | 475485 | 27/33 | 484.1 | 484 | 27 | 15.0 | (0.11, 0.30) | [17] |
| 14 | QA-PCZ | 471/480 | 29/34 | 339.1 | 482 | 29 | 17.5 | (0.11, 0.28) | [17] |
| 15 | QAD-Cz | 488/500 | 47/50 | 205 | 494 | 57 | 20.3 | (0.16, 0.47) | [18] |
| 16 | QAD-2Cz | 506/526 | 46/50 | 130 | 530 | 56 | 27.3 | (0.30, 0.65) | [18] |
| 17 | QAD-mTDPA | 586/587 | 55/62 | 269 | 589 | 67 | 26.3 | (0.55, 0.44) | [18] |
| 18 | QAO-PhCz | 460/- | 29/- | 40.4 | 467 | 36 | 14.0 | (0.13, 0.18) | [19] |
| 19 | QAOCz1 | 502/501 | 34/45 | 115.7 | 516 | 44 | 16.9 | (0.23, 0.66) | [20] |
| 20 | QAOCz2 | 500/500 | 29/41 | 16.4 | 504 | 43 | 19.4 | (0.18, 0.62) | [20] |
| 21 | QAOCz3 | 492/495 | 29/42 | 21.5 | 500 | 40 | 21.1 | (0.16, 0.57) | [20] |
| 22 | Cz-DiKTa | 495/502 | 54/54 | 196 | 511 | 62 | 24.9 | (0.24, 0.61) | [21] |
| 23 | Cz-Ph-DiKTa | 472/486 | 47/47 | 153 | 492 | 61 | 23.0 | (0.18, 0.50) | [21] |
| 24 | TMCz-DiKTa | 516/501 | 80/80 | 22 | 527 | 78 | 20.2 | (0.32, 0.60) | [21] |
| 25 | DMAc-DiKTa | 551/534 | 94/94 | 6.6 | 549 | 89 | 23.8 | (0.40, 0.57) | [21] |
| 26 | 3Cz-DiKTa | 534/539 | 53/53 | 286 | 547 | 54 | 24.4 | (0.39, 0.60) | [21] |
| 27 | 3TMCz-DiKTa | 617/577 | 110/110 | 3 | - | - | - | - | [21] |
| 28 | 3DMAc-DiKTa | 667/599 | 116/116 | 3.5 | - | - | - | - | [21] |
| 29 | TOAT | - | 16 | - | - | - | - | - | [22] |
| 30 | 1 | - | 70 | 650 | - | - | 0.14 | (0.23, 0.59) | [22] |
| 31 | 2 | - | 94 | 370 | - | - | 0.16 | (0.41, 0.56) | [22] |
| 32 | 3 | - | 84 | 4680 | - | - | 0.10 | (0.46, 0.54) | [22] |
| 33 | 4 | - | 75 | 3840 | - | - | 0.01 | (0.54, 0.45) | [22] |
| 34 | 5 | - | 45 | 2140 | - | - | 0.02 | (0.57, 0.43) | [22] |
| 35 | mBDPA-TOAT | 599/- | 37/- | 137 | 600 | 45 | 17.3 | (0.61, 0.39) | [23] |
| 36 | pBDPA-TOAT | 603/- | 48/- | 308 | 624 | 62 | 11.3 | (0.66, 0.34) | [23] |
| 37 | DMAC-TOAT | 656/- | 105/- | 37 | 616 | 104 | 1.5 | (0,59, 0.39) | [23] |
| 38 | 2,3-CZ | 449/- | 36/- | 436 | 458 | 52 | 6.3 | (0.15, 0.14) | [24] |
| 39 | 2,5-CZ | 459/- | 41/- | 619 | 463 | 48 | 22.3 | (0.13, 0.13) | [24] |
| 40 | 2,6-CZ | 497/- | 80/- | 28.1 | 489 | 66 | 21.2 | (0.15, 0.33) | [24] |
| 41 | 2,3-DPA | 496/- | 57/- | 373.1 | 503 | 54 | 11.7 | (0.17, 0.54) | [24] |
| 42 | 2,3-POA | 547/- | 92/- | 6.2 | 528 | 68 | 21.7 | (0.30, 0.62) | [24] |
| 43 | QA-1 | 434/457 | 31/43 | 655 | 455 | 49 | 17.1 | (0.14, 0.12) | [25] |
| 44 | QA-2 | 444/465 | 22/37 | 48 | 463 | 37 | 19.0 | (0.13, 0.14) | [25] |
| 45 | QA-3 | 485/523 | 64/73 | 307 | 515 | 67 | 18.6 | (0.26, 0.62) | [25] |
| 46 | Hel-DiDiKTa | 473/477 | 44/50 | 5.4 | - | - | - | - | [26] |
| 47 | CZCO | 430/- | 32/- | 392.8 | 432 | 44 | 26.9 | (0.154,0.051) | [27] |
| 48 | CZ2CO | 440/- | 16/- | 431.8 | 445 | 26 | 25.6 | (0.157,0.074) | [27] |
| 49 | DiKTa-LC | 487/512 | 33/50 | 155.5 | 492 | 51 | 13.6 | (0.22, 0.49) | [28] |
| 50 | 3TPA-DiKTa | 537/551 | 54/58 | 131 | 551 | 62 | 30.8 | (0.409, 0.577) | [29] |
| 51 | 3DPA-DiKTa | 597/617 | 47/56 | 323 | 613 | 60 | 16.7 | (0.633, 0.365) | [29] |
| 52 | BOQAO | 474/487 | 28/33 | 214 | 484 | 32 | 21.8 | (0.11, 0.37) | [30] |
| 53 | CzAO | 431/- | 36/- | 1340 | 444 | 43 | 8.6 | (0.114, 0.065) | [31] |
| 54 | MQAO | 447/- | 61/- | 1024 | 460 | 62 | 10.3 | (0.142, 0.139) | [31] |
| 55 | QPXO | 485/- | 76/- | 3595 | 486 | 67 | 7.1 | (0.166, 0.406) | [31] |
| 56 | QPO | 501/- | 86/- | 4430 | 510 | 82 | 15.3 | (0.237 , 0.529) | [31] |
| 57 | pe-QAO | 424/466 | 13/79 | - | 468 | 75 | 9.0 | - | [32] |
| 58 | [5]he-BQAO | 458/477 | 32/40 | - | 471 | 43 | 3.3 | - | [32] |
| 59 | [6]he-BQAO | 474/494 | 34/44 | - | - | - | - | - | [32] |
| 60 | hp-BQAO | 456/473 | 34/42 | 13.4 | 471 | 41 | 24.1 | - | [32] |
| 61 | DOBDiKTa | 445/460 | 27/37 | 43 | 458 | 38 | 17.4 | (0.14 , 0.12) | [33] |
| 62 | CZQ | 424/464 | 18/68 | 409 | 460 | 63 | 10.2 | (0.145, 0.125) | [34] |
| 63 | SFQ | 484/460 | 19/30 | 124 | 460 | 30 | 21.7 | (0.133, 0.106) | [34] |
| 64 | SOQ | 447/457 | 18/32 | 135 | 456 | 33 | 24.3 | (0.137, 0.104) | [34] |
| 65 | SSQ | 445/458 | 17/30 | 114 | 456 | 31 | 25.5 | (0.137, 0.105) | [34] |
| 66 | SSeQ | 446/456 | 18/34 | 131 | 460 | 35 | 22.2 | (0.144, 0.120) | [34] |
| 67 | Sym-DiDiKTa | 540/542 | 29/35 | 1700 | 543 | 36 | 9.8 | (0.359, 0.624) | [35] |
| 68 | Asym-DiDiKTa | 541/547 | 29/35 | 2100 | 548 | 56 | 19.9 | (0.397, 0.592) | [35] |
| 69 | PhCz-O-DiKTa | 478/502 | 39/57 | 653.5 | 497 | 58 | 20.0 | (0.21, 0.55) | [36] |
| 70 | PhCz-DiKTa | 490/528 | 55/72 | 103.8 | 524 | 72 | 22.0 | (0.31, 0.61) | [36] |
| 71 | MTDMQAO | 455/470 | 19/37 | 32 | 472 | 39 | 29.4 | (0.13, 0.21) | [37] |
| 72 | MBDMQAO | 469/480 | 20/28 | 540 | 480 | 27 | 18.9 | (0.12, 0.28) | [37] |
| 73 | S-DAO | 493/- | 24/- | 140 | 502 | 26 | 29.9 | (0.12, 0.62) | [38] |
| 74 | SS-DAO | 512/- | 21/- | 91 | 520 | 24 | 37.2 | (0.20, 0.73) | [38] |
| 75 | DDiKTa-F | 476/494 | 32/49 | 188 | 493 | 46 | 15.3 | (0.16, 0.50) | [39] |
| 76 | dBr-tBu-DiKTa | 464/483 | - | 310.2 | 480 | 54 | 21.2 | (0.16, 0.35 | [40] |
| 77 | tBr-DiKTa | 477/488 | - | 108.5 | - | - | - | - | [40] |
| **This**  **work** | **DPQAO-F** | **458/461** | **23/24** | **125.4** | **463** | **24** | **19.9** | **(0.134, 0.118)** | **TADF** |
|  |  |  |  |  | **461** | **36** | **32.7** | **(0.146, 0.117)** | **HF** |

**NMR Spectra**

**Figure S23.** ^1^H NMR spectrum of compound 3 in *d^6^*-DMSO solvent.

**Figure S24.** ^13^C NMR spectrum of compound 3 in *d^6^*-DMSO solvent.

**Figure S25.** ^1^H NMR spectrum of compound 5 in *d*-CD_2_Cl_2_ solvent.

**Figure S26.** ^13^C NMR spectrum of compound 5 in *d*-CD_2_Cl_2_ solvent.

**Figure S27.** ^1^H NMR spectrum of compound 9 in *d*-CD_2_Cl_2_ solvent.

**Figure S28.** ^13^C NMR spectrum of compound 9 in *d*-CD_2_Cl_2_ solvent.

**Figure S29.** ^1^H NMR spectrum of DPQAO-F in *d*-CD_2_Cl_2_ solvent.

**Figure S30.** ^13^C NMR spectrum of DPQAO-F in *d*-CD_2_Cl_2_ solvent.

**Figure S31.** ^1^H NMR spectrum of DPQAO-M in *d*-CD_2_Cl_2_ solvent.

**Figure S32.** ^13^C NMR spectrum of DQPAO-M in *d*-CD_2_Cl_2_ solvent.

**Figure S33.** MALDI-TOF mass spectrum of compound DPQAO-F.

**Figure S34.** MALDI-TOF mass spectrum of compound DPQAO-M.

**Reference**

[1] K.-H. Kim, J.-J. Kim, *Adv. Mater.* **2018**, *30*, 1705600.

[1] Gaussian 09, Revision A.01, M. J. Frisch, G. W. Trucks, H. B. Schlegel, G. E. Scuseria, M. A. Robb, J. R. Cheeseman, G. Scalmani, V. Barone, B. Mennucci, G. A. Petersson, H. Nakatsuji, M. Caricato, X. Li, H. P. Hratchian, A. F. Izmaylov, J. Bloino, G. Zheng, J. L. Sonnenberg, M. Hada, M. Ehara, K. Toyota, R. Fukuda, J. Hasegawa, M. Ishida, T. Nakajima, Y. Honda, O. Kitao, H. Nakai, T. Vreven, J. A. Montgomery, Jr., J. E. Peralta, F. Ogliaro, M. Bearpark, J. J. Heyd, E. Brothers, K. N. Kudin, V. N. Staroverov, R. Kobayashi, J. Normand, K. Raghavachari, A. Rendell, J. C. Burant, S. S. Iyengar, J. Tomasi, M. Cossi, N. Rega, J. M. Millam, M. Klene, J. E. Knox, J. B. Cross, V. Bakken, C. Adamo, J. Jaramillo, R. Gomperts, R. E. Stratmann, O. Yazyev, A. J. Austin, R. Cammi, C. Pomelli, J. W. Ochterski, R. L. Martin, K. Morokuma, V. G. Zakrzewski, G. A. Voth, P. Salvador, J. J. Dannenberg, S. Dapprich, A. D. Daniels, O. Farkas, J. B. Foresman, J. V. Ortiz, J. Cioslowski, and D. J. Fox, Gaussian, Inc., Wallingford CT, 2009.

[3] T. Lu, F. Chen, *J. Comput. Chem.* **2012**, *33*, 580.

[4] Z. Shuai, *Chin. J. Chem.* **2020**, *38*, 1223.

[5] M. J. Abraham, T. Murtola, R. Schulz, S. Páll, J. C. Smith, B. Hess, E. Lindahl, *SoftwareX* **2015**, *1–2*, 19.

[6] J. Wang, R. M. Wolf, J. W. Caldwell, P. A. Kollman, D. A. Case, *J. Comput. Chem.* **2004**, *25*, 1157.

[7] L. Martínez, R. Andrade, E. G. Birgin, J. M. Martínez, *J. Comput. Chem.* **2009**, *30*, 2157.

[8] T. Darden, D. York, L. Pedersen, *J. Chem. Phys.* **1993**, *98*, 10089.

[9] J. E. Field, T. J. Hill, D. Venkataraman, *J. Org. Chem.* **2003**, *68*, 6071.

[10] Y. Yuan, X. Tang, X.-Y. Du, Y. Hu, Y.-J. Yu, Z.-Q. Jiang, L.-S. Liao, S.-T. Lee, *Adv. Opt. Mater.* **2019**, *7*, 1801536.

[11] Q. Feng, Y. Qian, H. Wang, W. Hou, X. Peng, S. Xie, S. Wang, L. Xie, *Adv. Opt. Mater.* **2022**, *10*, 2102441.

[12] Y. Tsuchiya, S. Diesing, F. Bencheikh, Y. Wada, P. L. dos Santos, H. Kaji, E. Zysman-Colman, I. D. W. Samuel, C. Adachi, *J. Phys. Chem. A* **2021**, *125*, 8074.

[13] X. Li, Y.-Z. Shi, K. Wang, M. Zhang, C.-J. Zheng, D.-M. Sun, G.-L. Dai, X.-C. Fan, D.-Q. Wang, W. Liu, Y.-Q. Li, J. Yu, X.-M. Ou, C. Adachi, X.-H. Zhang, *ACS Appl. Mater. Interfaces* **2019**, *11*, 13472.

[14] D. Hall, S. M. Suresh, P. L. dos Santos, E. Duda, S. Bagnich, A. Pershin, P. Rajamalli, D. B. Cordes, A. M. Z. Slawin, D. Beljonne, A. Köhler, I. D. W. Samuel, Y. Olivier, E. Zysman-Colman, *Adv. Opt. Mater.* **2020**, *8*, 1901627.

[15] D. Sun, S. M. Suresh, D. Hall, M. Zhang, C. Si, D. B. Cordes, A. M. Z. Slawin, Y. Olivier, X. Zhang, E. Zysman-Colman, *Mater. Chem. Front.* **2020**, *4*, 2018.

[16] S.-N. Zou, C.-C. Peng, S.-Y. Yang, Y.-K. Qu, Y.-J. Yu, X. Chen, Z.-Q. Jiang, L.-S. Liao, *Org. Lett.* **2021**, *23*, 958.

[17] X. Qiu, G. Tian, C. Lin, Y. Pan, X. Ye, B. Wang, D. Ma, D. Hu, Y. Luo, Y. Ma, *Adv. Opt. Mater.* **2021**, *9*, 2001845.

[18] F. Huang, K. Wang, Y.-Z. Shi, X.-C. Fan, X. Zhang, J. Yu, C.-S. Lee, X.-H. Zhang, *ACS Appl. Mater. Interfaces* **2021**, *13*, 36089.

[19] S.-Y. Yang, S.-N. Zou, F.-C. Kong, X.-J. Liao, Y.-K. Qu, Z.-Q. Feng, Y.-X. Zheng, Z.-Q. Jiang, L.-S. Liao, *Chem. Commun.* **2021**, *57*, 11041.

[20] J.-F. Liu, S.-N. Zou, X. Chen, S.-Y. Yang, Y.-J. Yu, M.-K. Fung, Z.-Q. Jiang, L.-S. Liao, *Mater. Chem. Front.* **2022**, *6*, 966.

[21] S. Wu, W. Li, K. Yoshida, D. Hall, S. Madayanad Suresh, T. Sayner, J. Gong, D. Beljonne, Y. Olivier, I. D. W. Samuel, E. Zysman-Colman, *ACS Appl. Mater. Interfaces* **2022**, *14*, 22341.

[22] Y. Tsuchiya, Y. Ishikawa, S.-H. Lee, X.-K. Chen, J.-L. Brédas, H. Nakanotani, C. Adachi, *Adv. Opt. Mater.* **2021**, *9*, 2002174.

[23] X.-C. Fan, K. Wang, Y.-Z. Shi, J.-X. Chen, F. Huang, H. Wang, Y.-N. Hu, Y. Tsuchiya, X.-M. Ou, J. Yu, C. Adachi, X.-H. Zhang, *Adv. Opt. Mater.* **2022**, *10*, 2101789.

[24] X.-F. Luo, F.-L. Li, J.-W. Zou, Q. Zou, J. Su, M.-X. Mao, Y.-X. Zheng, *Adv. Opt. Mater.* **2021**, *9*, 2100784.

[25] H. Min, I. S. Park, T. Yasuda, *Angew. Chem. Int. Ed.* **2021**, *60*, 7643.

[26] J. M. dos Santos, D. Sun, J. M. Moreno-Naranjo, D. Hall, F. Zinna, S. T. J. Ryan, W. Shi, T. Matulaitis, D. B. Cordes, A. M. Z. Slawin, D. Beljonne, S. L. Warriner, Y. Olivier, M. J. Fuchter, E. Zysman-Colman, *J. Mater. Chem. C* **2022**, *10*, 4861.

[27] C. Cao, J.-H. Tan, Z.-L. Zhu, J.-D. Lin, H.-J. Tan, H. Chen, Y. Yuan, M.-K. Tse, W.-C. Chen, C.-S. Lee, *Angew. Chem. Int. Ed.* **2023**, *62*, e202215226.

[28] D. Chen, F. Tenopala-Carmona, J. A. Knöller, A. Mischok, D. Hall, S. Madayanad Suresh, T. Matulaitis, Y. Olivier, P. Nacke, F. Gießelmann, S. Laschat, M. C. Gather, E. Zysman-Colman, *Angew. Chem. Int. Ed.* **2023**, *62*, e202218911.

[29] S. Wu, A. Kumar Gupta, K. Yoshida, J. Gong, D. Hall, D. B. Cordes, A. M. Z. Slawin, I. D. W. Samuel, E. Zysman-Colman, *Angew. Chem. Int. Ed.* **2022**, *61*, e202213697.

[30] Y.-J. Yu, S.-N. Zou, C.-C. Peng, Z.-Q. Feng, Y.-K. Qu, S.-Y. Yang, Z.-Q. Jiang, L.-S. Liao, *J. Mater. Chem. C* **2022**, *10*, 4941.

[31] J.-W. Huang, Y.-C. Hsu, X. Wu, S. Wang, X.-Q. Gan, W.-Q. Zheng, H. Zhang, Y.-Z. Gong, W.-Y. Hung, P.-T. Chou, W. Zhu, *J. Mater. Chem. C* **2022**, *10*, 7866.

[32] Y. Wu, X. Liu, J. Liu, G. Yang, S. Han, D. Yang, X. Cao, D. Ma, Z. Bin, J. You, *Mater. Horiz.* **2023**, *10*, 3785.

[33] S. Wu, L. Zhang, J. Wang, A. Kumar Gupta, I. D. W. Samuel, E. Zysman-Colman, *Angew. Chem. Int. Ed.* **2023**, *62*, e202305182.

[34] Y.-J. Yu, Z.-Q. Feng, X.-Y. Meng, L. Chen, F.-M. Liu, S.-Y. Yang, D.-Y. Zhou, L.-S. Liao, Z.-Q. Jiang, *Angew. Chem. Int. Ed.* **2023**, *62*, e202310047.

[35] J. M. dos Santos, C.-Y. Chan, S. Tang, D. Hall, T. Matulaitis, D. B. Cordes, A. M. Z. Slawin, Y. Tsuchiya, L. Edman, C. Adachi, Y. Olivier, E. Zysman-Colman, *J. Mater. Chem. C* **2023**, *11*, 8263.

[36] T. Wang, A. K. Gupta, D. B. Cordes, A. M. Z. Slawin, E. Zysman-Colman, *Adv. Opt. Mater.* **2023**, *11*, 2300114.

[37] L. Chen, J.-H. Cai, Y.-J. Yu, Y.-K. Qu, S.-Y. Yang, S.-N. Zou, R.-H. Liu, D.-Y. Zhou, L.-S. Liao, Z.-Q. Jiang, *Sci. China Chem.* **2023**, DOI 10.1007/s11426-023-1669-9.

[38] L. Liang, C. Qu, X. Fan, K. Ye, Y. Zhang, Z. Zhang, L. Duan, Y. Wang, *Angew. Chem. Int. Ed.* **2023**, *n/a*, e202316710.

[39] S. Wu, Y.-N. Hu, D. Sun, K. Wang, X. Zhang, E. Zysman-Colman, *Chem. Commun.* **2023**, DOI 10.1039/D3CC05761E.

[40] H. G. Miranda-Salinas, J. Wang, A. Danos, T. Matulaitis, K. Stavrou, A. P. Monkman, E. Zysman-Colman, *J. Mater. Chem. C* **2023**, DOI 10.1039/D3TC04394K.
